# Overcoming Bcr-Abl T315I mutation by combination of GNF-2 and ATP competitors in an Abl-independent mechanism

**DOI:** 10.1186/1471-2407-12-563

**Published:** 2012-11-27

**Authors:** Mamduh Khateb, Nili Ruimi, Hazem Khamisie, Yousef Najajreh, Afsar Mian, Anna Metodieva, Martin Ruthardt, Jamal Mahajna

**Affiliations:** 1 Cancer Drug Discovery Program, Galilee Technology Center, Migal, P.O.Box 831, Kiryat Shmona, 11016, Israel; 2 Faculty of Pharmacy, Al-Quds University, Jerusalem-Abu Dies, Palestine; 3 Medizinische Klinik II/Abt. Hämatologie, Klinikum der Goethe-Universität, Theodor-Stern Kai 7, 60590, Frankfurt, Germany; 4 Department of Nutritional Sciences, Tel-Hai College, Kiryat Shmona, Israel

**Keywords:** Philadelphia chromosome, Bcr-Abl, “gatekeeper” mutation T315I, Allosteric inhibition, Abl kinase inhibitors

## Abstract

**Background:**

Philadelphia positive leukemias are characterized by the presence of Bcr-Abl fusion protein which exhibits an abnormal kinase activity. Selective Abl kinase inhibitors have been successfully established for the treatment of Ph (+) leukemias. Despite high rates of clinical response, Ph (+) patients can develop resistance against these kinase inhibitors mainly due to point mutations within the Abl protein. Of special interest is the ‘gatekeeper’ T315I mutation, which confers complete resistance to Abl kinase inhibitors. Recently, GNF-2, Abl allosteric kinase inhibitor, was demonstrated to possess cellular activity against Bcr-Abl transformed cells. Similarly to Abl kinase inhibitors (AKIs), GNF-2 failed to inhibit activity of mutated Bcr-Abl carrying the T315I mutation.

**Methods:**

Ba/F3 cells harboring native or T315I mutated Bcr-Abl constructs were treated with GNF-2 and AKIs. We monitored the effect of GNF-2 with AKIs on the proliferation and clonigenicity of the different Ba/F3 cells. In addition, we monitored the auto-phosphorylation activity of Bcr-Abl and JAK2 in cells treated with GNF-2 and AKIs.

**Results:**

In this study, we report a cooperation between AKIs and GNF-2 in inhibiting proliferation and clonigenicity of Ba/F3 cells carrying T315I mutated Bcr-Abl. Interestingly, cooperation was most evident between Dasatinib and GNF-2. Furthermore, we showed that GNF-2 was moderately active in inhibiting the activity of JAK2 kinase, and presence of AKIs augmented GNF-2 activity.

**Conclusions:**

Our data illustrated the ability of allosteric inhibitors such as GNF-2 to cooperate with AKIs to overcome T315I mutation by Bcr-Abl-independent mechanisms, providing a possibility of enhancing AKIs efficacy and overcoming resistance in Ph+ leukemia cells.

## Background

Philadelphia positive leukemias are hematological malignancies caused by a chromosomal rearrangement that generates a fusion protein, Bcr–Abl, with deregulated tyrosine kinase activity. Imatinib, which targets the ATP-binding site, is effective in the early stage of the treatment of Ph-positive patients, but advanced-stage patients may relapse as a result of the emergence of point mutations within the Bcr–Abl. Two recently approved drugs, Nilotinib [1] and Dasatinib [2] inhibit the activity of mutated Bcr-Abl that is refractory to Imatinib except the ‘gatekeeper’ T315I mutation, which is situated in the middle of the ATP-binding cleft [3].

Allosteric kinase inhibitors hold promise for revealing unique features of kinases that may not be apparent using conventional ATP-competitive inhibitors. Thus, using an unbiased cellular screening approach, GNF-2, a non-ATP-competitive inhibitor, has been identified and shown to demonstrate cellular activity against Bcr-Abl transformed cells [4]. The exquisite selectivity of GNF-2 is due to the finding that it targets the myristate binding site located near the C-terminus of the Abl kinase domain, as demonstrated by genetic approaches, solution NMR, X-ray crystallography, mutagenesis and hydrogen exchange mass spectrometry [5]. GNF-2, like myristate, is able to induce and/or stabilize the clamped inactive conformation of Abl, analogous to the SH2-Y527 interaction of Src [6]. Crystallography study revealed that GNF-2 replaces the myristoylated peptide in the crystals [5]. As expected, most of the interactions between GNF-2 and the protein are hydrophobic. Mutations of three residues near the mouth of the myristate-binding site (C464Y, P465S and V506L) were reported to cause resistance to the binding of GNF-2, presumably for steric reasons. The myristate-binding-site mutant, E505K, was inhibited by Imatinib and Nilotinib, but not by GNF-2, arguing that GNF-2 targets the myristoyl pocket [5].

In this report we showed that GNF-2 cooperated with the Abl kinase inhibitors (AKIs), Imatinib, Nilotinib and Dasatinib, in inhibiting clonigenicity of Bcr-Abl T315I transformed Ba/F3 cells. Interestingly, activity against T315I mutation was Bcr-Abl independent. Furthermore, GNF-2 and AKIs also cooperated to inhibit JAK2 phosphorylation in Ba/F3 carrying T315I mutation.

## Materials and methods

### Cell lines and cell cultures

Ba/F3 cells expressing Bcr-Abl constructs or activated JAK2 (V617F) were previously described [7] and grown in RPMI 1640 with 2 mM L-glutamine supplemented with 10% fetal bovine serum. Penicillin at 100 U/ml, and streptomycin at 100 μg/ml, was added to the culture media. SupB15, a Ph+ ALL B cell (ATCC, Rockville, MD) was grown in RPMI 1640 containing 2 mM L-glutamine, 20% FBS, 100 U/ml penicillin and 100 μg/ml streptomycin. All cell lines were grown at 37°C in a humidified atmosphere with 5% CO2.

### Cellular Bcr-Abl auto-phosphorylation and immune-blotting

Ba/F3 cells expressing the native or the T315I mutated Bcr-Abl protein (4 x 10^5^ cells/ml) were treated with Abl kinase inhibitors (AKIs), GNF-2, combinations of GNF-2 and AKIs and DMSO for 1 h. Cells were collected, washed once with cold PBS, and lysed as previously describe [7]. Cell lysate supernatants (40 μg protein) were resolved on 8% SDS-polyacrylamide gel electrophoresis, transferred to nitrocellulose membranes, and analyzed by immune-blotting with Anti-phospho-c-Abl (Tyr245), Anti-phospho-STAT5α (Tyr694) and anti-phospho JAK2 (Tyr1007/1008) antibodies (Cell Signaling Technology, USA). The phosphorylated level of Bcr-Abl protein was compared to total Abl or α-tubulin that were detected using Anti-c-Abl and Anti-α-tubulin antibodies (Santa Cruz Biotechnology, USA). Quantitative analysis of the protein bands detected by Western blot was carried out using Tina software 2.0. Analyses of pSTATα pBcr-Abl and pJAK2 levels are given as folds of the sample values versus the α -tubulin values used as a loading control.

### Trypan blue exclusion assay

Ba/F3 cells containing Bcr-Abl constructs were plated (4x 10^5^ cells/well) in six-well plates, with each well containing 3 ml medium. After 24 h, cells were treated with the appropriate agents. Solvent-treated samples were incubated with 1% DMSO. Seventy-two hours later, the cells were collected, stained with 0.4% trypan blue solution (1:1), and counted using a hemocytometer to determine IC_50_ values.

### Colony-forming assay

A colony-forming assay was performed as previously described [7]. Briefly, cells (1 x 10^4^) in 1 ml RPMI/10% FBS medium were diluted in 1 ml of 0.6% agar to give a final agar concentration of 0.3% agar. The cell-agar mixture was poured on top of a hardened agar base in wells of 12-well plates and allowed to solidify. Once the top layer solidified, 1 ml of medium containing different treatments was placed on top to keep the agar moist. The cells were grown at 37°C in a 5% CO2 humidified atmosphere until colonies were visible (2 weeks). The plates were stained for 4 h with 5 mg/ml 3-(4,5-dimethylthiazol-2-yl)-2,5-diphenyltetrazolium bromide (MTT), and the dye was extracted with 1 ml solubilization buffer (20% sodium dodecyl sulfate [SDS], 50% N,N-dimethyl-formamide, 25 mM HCL) for 24 h. The optical density was measured at 570 nm wavelength with a reference wavelength of 630 nm.

### Statistical analysis

Statistical analysis was performed using Student’s *t*-test, with significant values set at *P<0.05 or **P<0.005.

## Results

**Figure 1:**
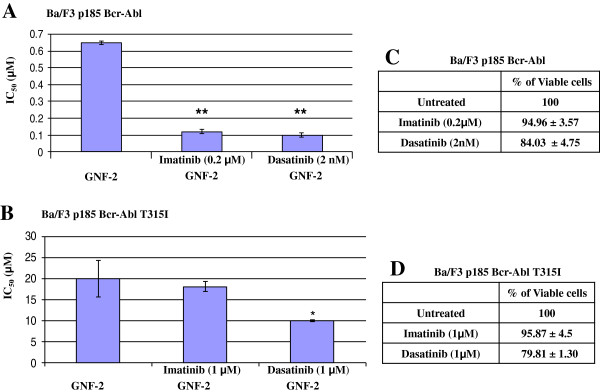
**Cooperation between GNF-2 and suboptimal concentration of Imatinib and Dasatinib in inhibiting proliferation of Bcr-Abl transfected Ba/F3 cells.** Ba/F3 cells, transfected with native p185 Bcr-Abl (**A**,**C**) and T315I mutated form (**B**,**D**) were treated with increasing GNF-2 concentrations and with Imatinib at 0.2 μM (**A**) and 1 μM (**B**), or with dasatinib at 2 nM (A) and 1 μM (B). In (**C**) and (**D**) percentage of viable Ba/F3 p185 Bcr-Abl (**C**) and Ba/F3 p185 Bcr-Abl T315I (**D**) cells treated with suboptimal concentration of Imatinib and Dasatinib for 72 h as described in Materials and Methods. IC_50_ values +/− SD were calculated and blotted. Data are of representative experiment. Experiment was repeated 3 times with similar outcomes. P-values; * p<0.05, ** p<0.005.

Since the efficacy of Imatinib and other ATP-competitors are moderate in ALL patients, and based on our previous work showing that the allosteric inhibitor GNF-2 is less active on p185 Bcr-Abl [8]; we wanted to seek efficacy improvement by combination of ATP-competitor with allosteric inhibitors, such as GNF-2. Initially, we tested the influence of suboptimal Imatinib (0.2 μM) and Dasatinib (2 nM) concentrations on the anti-proliferative activity of GNF-2 using Ba/F3 p185 Bcr-Abl transfected cells. Imatinib (0.2 μM) and Dasatinib (2 nM) exhibited only marginal effect on proliferation of Ba/F3 carrying the native Bcr-Abl (Figure 1C). Furthermore, 1 μM of Imatinib and Dasatinib failed to significantly inhibit Ba/F3 cells carrying the T315I mutation (Figure 1D). The presence of suboptimal concentrations of Imatinib (0.2 μM) and Dasatinib (2nM) reduced the IC_50_ of GNF-2 from 0.65 to 0.12 and 0.1 μM, respectively, when cells were treated for 72 h (Figure 1A). In addition, in Ba/F3 transfected with p185 Bcr-Abl T315I mutation, which are refractory to Imatinib, Dasatinib, and GNF-2, presence of Imatinib and Dasatinib at 1 μM reduced the IC_50_ of GNF-2 from 20 to 18 and 10 μM, respectively (Figure 1B).

Interestingly, no cooperation was observed in inhibiting the parental Ba/F3 cells by GNF-2 and AKIs (Additional file 1: Figure S1 and S2).

### Cooperation between GNF-2 and Abl Kinase Inhibitors (AKIs) in inhibiting clonigenicity of BaF3/p185 Bcr-Abl T315I cells

Anchorage-independent growth of cells is a typical characteristic of the tumorigenicity of cancer cells in vitro [9]. Thus, we tested the ability of Abl kinase inhibitors (AKIs) to affect clonigenicity of Ba/F3 p185 Bcr-Abl T315I in the presence of GNF-2.

**Figure 2:**
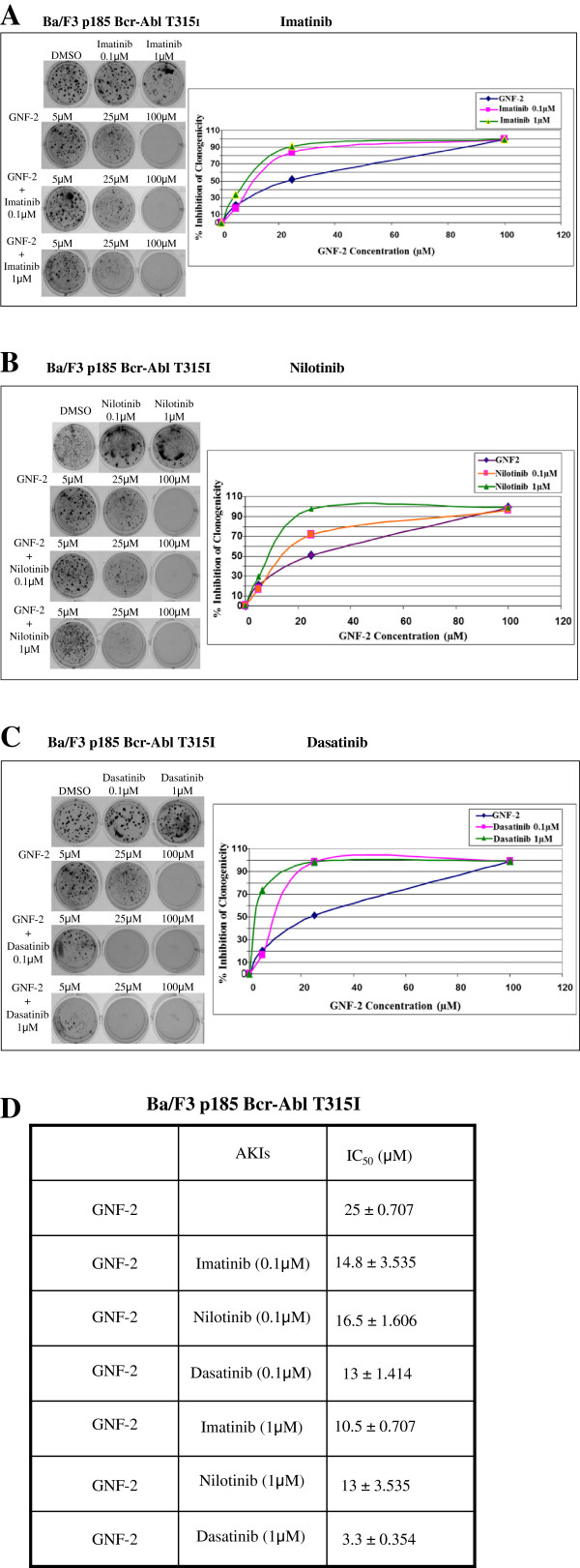
**Cooperation between GNF-2 and AKIs in clonigenicity inhibition of Ba/F3 p185 Bcr-Abl T315I cells.** Clonigenicity assay was performed as described in Materials and Methods. Ba/F3 transfected cells were treated with GNF-2 at 5, 25 and 100 μM, in the presence of 0.1 μM and 1 μM of Imatinib (**A**), Nilotinib (**B**) and Dasatinib (**C**). IC_50_ values +/− SD were calculated and listed (**D**).

Data presented in Figure 2 illustrated that GNF-2 was active in substantially inhibiting clonigenicity of Ba/F3 p185 Bcr-Abl T315I at 100 μM (Figure 2A-2C). The calculated IC_50_ was 25 μM (Figure 2D). As expected, clonigenicity of Ba/F3 p185 Bcr-Abl T315I was not affected by all AKIs used at 100 nM or 1000 nM (Figure 2A-2C). Addition of AKIs at 10 nM with various concentration of GNF-2 did not sensitize Ba/F3 p185 Bcr-Abl T315I cells to GNF-2-dependent clonigenicity inhibition (data not shown). However, presence of AKIs at 100 nM showed a marginal cooperation between GNF-2 and Imatinib and Nilotinib (Figure 2A-2B) and a greater cooperation with Dasatinib (Figure 2C). The IC_50_ of GNF-2 was reduced from 25 μM to 14.8 μM, 16.5 μM and 13 μM when Imatinib, Nilotinib and Dasatinib at 100nM were added, respectively (Figure 2D). When AKIs at 1 μM were used, we observed a more noticeable cooperation with GNF-2. Presence of Imatinib (Figure 2A), Nilotinib (Figure 2B) and Dasatinib (Figure 2C) reduced the IC_50_ of GNF-2 to 10.5, 13, and 3.3 μM, respectively (Figure 2D). Interestingly, Dasatinib was the most efficient AKI in cooperation with GNF-2 in inhibiting clonigenicity of Ba/F3 cells containing the T315I mutated Bcr-Abl.

Interestingly, no cooperation was observed in inhibiting the clonigenicity of the parental Ba/F3 cells by GNF-2 and AKIs (Additional file 1: Figure S3).

### Cooperation between GNF-2 and Abl Kinase Inhibitors (AKIs) in inhibiting auto-phosphorylation of Bcr-Abl

**Figure 3:**
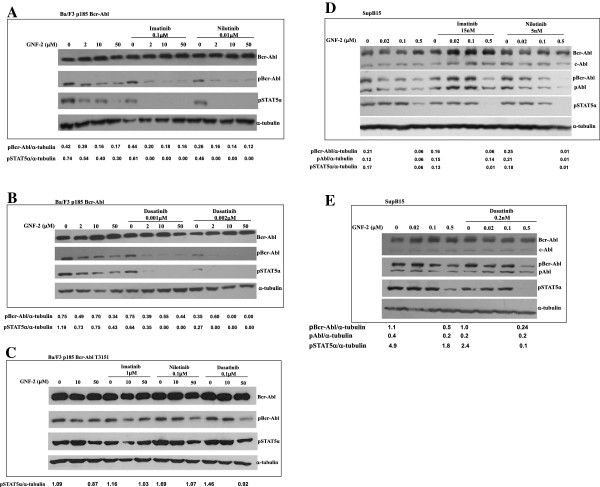
**Cooperation between GNF-2 and AKIs in inhibition phosphorylation of Bcr-Abl and STAT5α.** Ba/F3, a laboratory model of Ph + cells carrying native Bcr-Abl (**A** and **B**), T315I mutated Bcr-Abl (**C**) and SupB15, a patient derived Ph+ ALL cell line (**D**-**E**) were treated with various concentrations of GNF-2 in the presence of AKIs, as indicated in each figure. Phosphorylation levels of Bcr-Abl, c-Abl (D-E) and STAT5α were measured by immuno-blotting. The α-tubulin protein was used as loading control. Relative values of pBcr-Abl, pAbl and pSTAT5α relative to α-tubulin are shown.

Next, we tested whether the cooperation between GNF-2 and AKIs in proliferation and clonigenicity inhibition of Bcr-Abl transfected Ba/F3 cells is mediated by interfering with Bcr-Abl activity. Results presented in Figure 3 demonstrated, as expected, that GNF-2 is active in inhibiting p185 Bcr-Abl auto-phosphorylation (Figure 3A and 3B). Moderate cooperation was observed when 0.1 μM Imatinib and 0.01 μM Nilotinib was used and resulted in marginally reducing the amount of pBcr-Abl compared to control samples (Figure 3A). However, a significant cooperation was seen using Dasatinib (0.001 and 0.002 μM) (Figure 3B). Levels of pBcr-Abl were partially reduced (50%) when 50 μM of GNF-2 were used (Figure 3B). However, presence of 0.001 μM and 0.002 μM of Dasatinib inhibited completely pBcr-Abl levels (Figure 3B). Levels of pSTAT5α were also marginally reduced when GNF-2 was applied in the presence of Imatinib, Nilotinib (Figure 3A) and significantly reduced in the presence of Dasatinib (Figure 3B).

### Cooperation between GNF-2 and Abl Kinase Inhibitors (AKIs) in inhibiting auto-phosphorylation of the mutated T315I Bcr-Abl

Possible cooperation between GNF-2 and AKIs in modulating the phosphorylation of Bcr-Abl T315I protein was also tested using cell-based auto-phosphorylation assay. Results presented in Figure 3C showed that GNF-2 and AKIs failed to significantly interfere neither with p185 Bcr-Abl auto-phosphorylation nor with STAT5α phosphorylation in Ba/F3 cells carrying the mutated T315I form of Bcr-Abl (Figure 3C). Presence of AKIs only marginally enhanced GNF-2 activity in affecting Bcr-Abl auto-phosphorylation and STAT5α phosphorylation (Figure 3C).

### Cooperation between GNF-2 and Abl Kinase Inhibitors (AKIs) in inhibiting Bcr-Abl auto-phosphorylation in patient derived SupB15 cell lines

Sub-optimal concentrations of AKIs were also used to monitor cooperation with GNF-2 in SupB15, a patient derived Ph+ ALL cell line. Data presented in Figure 3D showed that GNF-2 at 0.5 μM is partially active in inhibiting Bcr-Abl auto-phosphorylation and STAT5α phosphorylation. Presence of Sub-optimal concentrations of Nilotinib (5 nM) demonstrated good cooperation in inhibiting Bcr-Abl auto-phosphorylation. Interestingly, we noticed greater activity on Bcr-Abl compared to endogenous c-Abl when Imatinib was used alongside GNF-2 (Figure 3D). Furthermore, cooperation was more profound in inhibiting STAT5α phosphorylation (Figure 3D) as evident in the combination of Imatinib and GNF-2.

Cooperation was also evident in the presence of low Dasatinib concentration (0.2 nM) and this concentration was sufficient to augment GNF-2 activity and to cause a significant inhibition of Bcr-Abl auto-phosphorylation and STAT5α phosphorylation when combined with 0.5 μM of GNF-2 (Figure 3E). Presence of 0.2 nM Dasatinib alongside of 0.5 μM GNF-2 reduced the relative pBcr-Abl, pAbl and pSTAT5α by 4.1, 1 and 24 fold, respectively (Figure 3E).

Results in Figure 3 demonstrated that Abl proteins, endogenous c-Abl and the chimeric Bcr-Abl, showed different degrees of sensitivity to GNF-2 alone, and in combinations with AKIs. GNF-2 alone, or with AKIs, exhibited good inhibitory activity on Bcr-Abl auto-phosphorylation, and only a marginal inhibition of the endogenous c-Abl auto-phosphorylation (Figure 3A3E). Our data are consistent with observation made by Choi et al., 2009 showing that GNF-2 inhibits the kinase activity of non-myristoylated c-Abl (Bcr-Abl) more potently than that of myristoylated c-Abl (endogenous Abl) [10].

### Cooperation between GNF-2 and Abl Kinase Inhibitors (AKIs) in inhibiting JAK2 activity

Our data showed that cooperation of GNF-2 and Abl kinase inhibitors (AKIs) on Ba/F3 p185 Bcr-Abl exhibited a more profound activity on STAT5α phosphorylation than Abl auto-phosphorylation (Figure 3A-3E). STAT5α phosphorylation is regulated by Bcr-Abl and JAK2, thus we investigated the effect of GNF-2, in combination with AKIs, on JAK2 activity.

**Figure 4:**
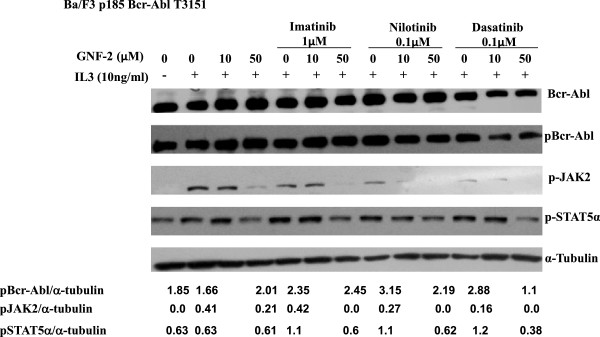
**Effect of GNF-2 and AKIs on pBcr-Abl, pSTAT5α and pJAK2 in Ba/F3p185 Bcr-Abl T315I exposed to IL-3=(10 ng/ml).** Ba/F3 cells carrying p185 Bcr-Abl T315I were exposed to IL-3 (10 ng/ml) and GNF2 (10 μM and 50 μM) alone or in combination with Imatinib (1 μM), Nilotinib (0.1 μM) and Dasatinib (0.1 μM) and levels of Bcr-Abl, pBcr-Abl, pJAK2, pSTAT5α and α-tubulin were determined by immune-blotting as previously described. Relative values of pBcr-Abl, pJAK2 and pSTAT5α relative to α-tubulin are shown.

The Ba/F3 Bcr-Abl T315I cells were treated with IL-3 and GNF-2/AKIs, and levels of pJAK2 and pSTAT5α were monitored. Results presented in Figure 4 demonstrated that levels of pJAK2 significantly increased in the presence of IL-3. Treatment of Ba/F3 Bcr-Abl T315I cells with GNF-2 caused a significant inhibition of pJAK2 at the higher concentration used (50 μM). Presence of AKIs augmented the inhibition of pJAK2 arguing that GNF-2 and AKIs cooperated in mediating inhibition of pJAK2. Interestingly, phosphorylation levels of STAT5α were constitutively high and were further increased in the presence of IL-3, while presence of GNF-2 or GNF-2/AKI reduced pSTA5α to the levels obtained in the absence of IL-3, arguing that STAT5α phosphorylation is mediated mainly by Bcr-Abl and is less dependent on JAK2 activation, and suggesting that GNF-2 and AKIs are not direct inhibitors of STAT5α.

**Figure 5:**
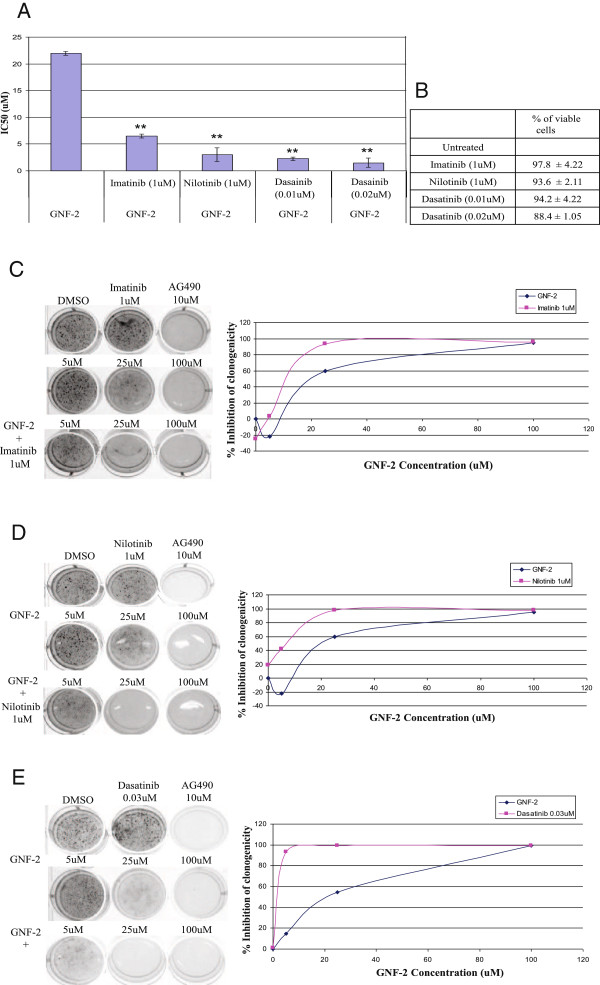
**Cooperation between GNF-2 and AKIs in inhibiting proliferation and clonigenicity of Ba/F3 JAK2 V617F cells.** (**A**). Effect of GNF-2 and sub-optimal concentrations of AKIs on the observed IC_50_s using Ba/F3 JAK2 V617F cells. (**B**). Percentage of viable cells (+/− SD) treated for 72 h with AKIs at the indicated concentrations. Effect of GNF-2 with Imatinib (**C**), Nilotinib (**D**) and Dasatinib (**E**) on the anchorage-independent growth of Ba/F3 JAK2 V617F cells was evaluated using a colony forming assay, and the concentrations inhibiting clonigenicity by 50% (IC_50_) were calculated. P-values * p<0.05, ** p<0.005.

Cooperation between GNF-2 and AKIs in inhibition proliferation of Ba/F3 cells transfected with activated JAK2 (JAK2 V617F) was also monitored. Data presented in Figure 5 showed that Imatinib and Nilotinib at 1 μM exhibited no inhibition of Ba/F3 JAK2 V617F proliferation (Figure 5B). In addition, GNF-2 inhibited proliferation of Ba/F3 JAK2 V617F with IC_50_ of 22 μM (Figure 5A). Presence of 1 μM of Imatinib and Nilotinib reduced the IC_50_ to 6.5 μM and 3 μM, respectively (Figure 5A). Proliferation of Ba/F3 JAK2 V617F was significantly inhibited by 1 μM Dasatinib (data not shown). Thus, we used sub-optimal concentration of Dasatinib, 10 nM and 20 nM, to assess potential cooperation with GNF-2 (Figure 5B). Presence of Dasatinib at 10 nM or 20nM with GNF-2 inhibited proliferation of Ba/F3 JAK2 V617F with IC_50_ of 2.25 μM and 1.5 μM, respectively (Figure 5A).

Next, we evaluated whether the GNF-2/AKIs cooperation also affects clonigenicity of Ba/F3 carrying the JAK2 V617F mutation. Data presented in Figure 5C-E showed that Imatinib, Nilotinib, and GNF-2 exhibited a minimal effect on clonigenicity of Ba/F3 JAK2 V617F cells. In contrast, AG490 (10 μM), a JAK2 inhibitor, significantly inhibited the clonigenicity of Ba/F3 JAK2 V617F cells (Figure 3C-3E). The IC_50_ of GNF-2 was 22.5 μM, and presence of 1 μM of either Imatinib or Nilotinib with GNF-2 significantly reduced the IC_50_ to 15.5 and 8 μM, respectively (Figure 5C and 5D). Although 1 μM of Dasatinib was active in inhibiting the clonigenicity of Ba/F3 JAK2 V617F cells (data not shown), we found that concentrations below 30 nM have a marginal effect on clonigenicity of the above cells (data not shown). Thus, we tested potential cooperation between GNF-2 and 30 nM Dasatinib. Results present in Figure 5E demonstrated a significant cooperation between Dasatinib and GNF-2 leading to the reduction of IC_50_ from 22.5 μM to 2.5 μM.

## Discussion and conclusion

Previously, the Abl allosteric inhibitor, GNF-2, was shown to cooperate with Imatinib and Nilotinib in inhibiting Bcr-Abl [5]. Furthermore, GNF-2 was also reported to cooperate with oligomerization inhibitors in inhibiting Bcr-Abl, as well as in overcoming T315I resistance [11]. Our results corporate the reported data showing a cooperation between Abl allosteric inhibitor, GNF-2, and Abl ATP competitors (Imatinib and Nilotinib), in inhibiting the proliferation of Ba/F3 cells carrying the native or the T315I mutated Bcr-Abl. However, no cooperation was observed between GNF-2 and AKIs in controlling the proliferation and clonigenicity of the parental Ba/F3 cells (Additional file 1). In this report we also demonstrated that Dasatinib, an Abl/Src dual inhibitor, is capable of cooperating with GNF-2 in inhibiting the proliferation of Ba/F3 cells carrying the native or the T315I mutated Bcr-Abl. In fact, our data showed that the cooperation with Dasatinib was more potent than the one observed with Imatinib or Nilotinib. Furthermore, cooperation between GNF-2 and AKIs was also evident in inhibiting clonigenicity of Ba/F3 cells carrying the T315I mutation of Bcr-Abl. Presence of 1 μM of the AKIs reduced the IC_50_ of GNF-2 from 25 μM to 10.5 μM, 13 μM, and 3.5 μM when Imatinib, Nilotinib and Dasatinib were used, respectively. Although we did not closely investigate the nature of the cooperation between AKIs and GNF-2 in the current study, it seems that GNF-2 and Dasatinib cooperated in a synergistic manner which is consistent with Mian et al., 2012 finding who demonstrated a synergistic relation between GNF-2 and Dasatinib [12].

Weisberg et al., 2010 showed that HG-7-85-01, ATP competitor, is capable of inhibiting the Bcr-Abl-T315I gatekeeper mutant. Furthermore, HG-7-85-01 was found to have additive effect in in-vitro and in vivo models in a Bcr-Abl-dependent fashion [1]. Our study utilized GNF-2, and other AKIs that are not active in inhibiting the T315I mutation, resulting in the inhibition of proliferation and clonigenicity of the T315I cells via a mechanism that is not dependent on Bcr-Abl, but rather on an alternative or downstream pathways.

Next, we explored the molecular mechanism responsible for the observed cooperation. Initially, we monitored the cooperation between GNF-2 and suboptimal concentration of AKIs in inhibiting the native Bcr-Abl auto-phosphorylation. Our results, presented in Figure 3, showed a moderate cooperation in inhibiting the phosphorylation of native Bcr-Abl and STAT5α. Our data are in agreement with data generated using flow cytometry analysis illustrating that GNF-5, a GNF-2 analog, cooperated with Nilotinib to inhibit STAT5α phosphorylation [5]. In contrast, only minimal cooperation was seen when Ba/F3 cells carrying the T315I Bcr-Abl was used. These results illustrated that the cooperative inhibition of Ba/F3 cells' proliferation and clonigenicity is not mediated by the Bcr-Abl protein, and that probably the two kinase inhibitors target downstream or alternative signaling pathways that control the growth of these cells.

Results shown in Figures 4 and 5 illustrated that JAK2 is also targeted by GNF-2, however, with reduced potency, consistent with the presence of a myristate binding pocket (MBP) within the JAK2 kinase (data not shown). Moreover, presence of AKIs augmented the inhibitory effect exerted by GNF-2. Interestingly, combination of GNF-2 and Dasatinib was the most efficient combination in inhibiting JAK2 phosphorylation.

Our data are also consistent with findings made by Nelson et al., 2011 showing that inhibitors of alternate pathways, such as STAT5α inhibitors, might be utilized as an effective therapy for Ph+ leukemia carrying native and T315I mutated Bcr-Abl [13]. The enhanced activity of the combination of GNF-2 with Dasatinib, a dual src/Abl kinase inhibitor, might be due to the inhibitory activity of Dasatinib on Src kinase which is involved in STAT5α phosphorylation [14].

In conclusion, our data provide evidence for cooperation between GNF-2 and AKIs in inhibiting proliferation and clonigenicity of Ba/F3 cells carrying T315I mutated Bcr-Abl construct. In our experimental system we used a laboratory model of p190 Bcr–Abl, a variant commonly found in acute lymphocytic leukemia (ALL) that typically responds only transiently to AKIs therapy, arguing that ALL patients may benefits from such combination. Cooperation between GNF-2 and AKIs was not mediated by Bcr-Abl protein inhibition, since the phosphorylation levels of Bcr-Abl and STA5α were not affected in Ba/F3 harboring T315I mutated Bcr-Abl. In summary, we showed that drug combination of allosteric inhibitors and AKIs, Dasatinib in particular, allows overcoming resistance in Ph+ leukemia cells, including cells harboring the T315I mutation.

## Competing interests

The authors declare that they have no competing interests.

## Authors’ contribution

MK carried out the studies on transduced Ba/F3 cells performing proliferation and clonigenicity studies. NR performed the auto-phosphorylation experiments and participated in drafting the manuscript. HK conducted the experiment of the parental Ba/F3 grown in the presence of IL-3 and experiments of Ba/F3 carrying JAK2 construct .AAM and AM generated the different Ba/F3 cells carrying the various Bcr-Abl constructs and participated in drafting the manuscript. MR and YN participated in the design of the study and performed the statistical analysis. JM conceived the study, supervised it and wrote the manuscript. All authors read and approved the final manuscript.

## Pre-publication history

The pre-publication history for this paper can be accessed here:

http://www.biomedcentral.com/1471-2407/12/563/prepub

## Supplementary Material

Additional file 1 **Effect of GNF-2 and Abl kinase Inhibitors (AKIs) on the proliferation and clonigenicity of Ba/F3 cells.Figure S1**: Effect of Imatinib and Dasatinib on the proliferation of Ba/F3 (Blue) and Ba/F3 p185 Bcr-Abl (Red) cells. The cells supplemented with 10 ng/ml of IL-3, were grown for 72 h in the presence of different Imatinib and Dasatinib concentrations. Cells were counted and percent of inhibition was calculated in relation to the solvent (0.5% DMSO) treated samples. Experiments were carried out in duplicates and repeated twice with comparable outcome. **Figure S2**: Cooperation between GNF-2 with Imatinib and Dasatinib in regulating proliferation of Ba/F3 cells. Ba/F3 supplemented with 10 ng/ml of IL-3 were grown for 72 h in the presence of various GNF-2 concentration (0.1, 0.5, 2.5, 5, 25 and 125 μM) alone or in combination with Imatinib (1 μM) and Dasatinib (50 nM). After 72 h incubation, cells were counted and percent of inhibition was calculated in relation to the solvent (0.5% DMSO) treated samples. Experiments were carried out in duplicates and repeated twice with comparable outcome. **Figure S3**: Effect of GNF-2 and AKIs on the clonigenicity of Ba/F3 cells. Ba/F3 cells grown on soft agar were treated with solvent (DMSO 0.5%; Un), Imatinib (1 μM), Dasatinib (1 μM), and GNF-2 (5 μM-100 μM) alone or in the presence of either 1 μM Imatinib or 1 μM Dasatinib. Experiments were carried out in duplicates and repeated twice with comparable outcome. Click here for file
