# Supplementary material for: Overcoming Bcr-Abl T315I mutation by combination of GNF-2 and ATP competitors in an Abl-independent mechanism
Source: BMC Cancer. 2012 Nov 27;12:563. doi: 10.1186/1471-2407-12-563 (PMC3561207; doi:10.1186/1471-2407-12-563)
Supplement: Additional file 1 — Effect of GNF-2 and Abl kinase Inhibitors (AKIs) on the proliferation and clonigenicity of Ba/F3 cells.Figure S1: Effect of Imatinib and Dasatinib on the proliferation of Ba/F3 (Blue) and Ba/F3 p185 Bcr-Abl (Red) cells. The cells supplemented with 10 ng/ml of IL-3, were grown for 72 h in the presence of different Imatinib and Dasatinib concentrations. Cells were counted and percent of inhibition was calculated in relation to the solvent (0.5% DMSO) treated samples. Experiments were carried out in duplicates and repeated twice with comparable outcome. Figure S2: Cooperation between GNF-2 with Imatinib and Dasatinib in regulating proliferation of Ba/F3 cells. Ba/F3 supplemented with 10 ng/ml of IL-3 were grown for 72 h in the presence of various GNF-2 concentration (0.1, 0.5, 2.5, 5, 25 and 125 μM) alone or in combination with Imatinib (1 μM) and Dasatinib (50 nM). After 72 h incubation, cells were counted and percent of inhibition was calculated in relation to the solvent (0.5% DMSO) treated samples. Experiments were carried out in duplicates and repeated twice with comparable outcome. Figure S3: Effect of GNF-2 and AKIs on the clonigenicity of Ba/F3 cells. Ba/F3 cells grown on soft agar were treated with solvent (DMSO 0.5%; Un), Imatinib (1 μM), Dasatinib (1 μM), and GNF-2 (5 μM-100 μM) alone or in the presence of either 1 μM Imatinib or 1 μM Dasatinib. Experiments were carried out in duplicates and repeated twice with comparable outcome. [file 1471-2407-12-563-S1.docx]

# Effect of GNF-2 and Abl kinase Inhibitors (AKIs) on the proliferation and clonigenicity of Ba/F3 cells

We tested the ability of Imatinib and Dasatinib to affect the growth of the parental Ba/F3 cells grown in the presence of IL-3 (10 ng/ml). Cells were treated with increasing concentration of Imatinib and Dasatinib for 72 hrs. Percentage of growth inhibition was calculated relative to solvent-treated samples. Data shown in Figure S1 indicated that Imatinib at 5μM had very little effect on growth of Ba/F3 cells; more than 90% of the cells were viable. In contrast, Dasatinib was more potent in inhibiting the growth of Ba/F3 cells. Presence of Dasatinib at 2.5 μM caused a decrease in cell survival to 65%.

Interestingly, Dasatinib at 50nM and Imatinib at 1μM caused minimal proliferation inhibition, of less than 10%, and concentrations were selected to verify cooperation with GNF-2, in affecting proliferation and survivals of Ba/F3 cells.

Data presented in figure S2 showed that Imatinib (1 μM) and Dasatinib (50nM) failed to cooperate with GNF-2 in affecting proliferation of Ba/F3 cells. The calculated IC_50_ of cells treated with GNF-2, in the absence or presence of Imatinib and Dasatinib, was almost identical, of 30 +/- 0.2 μM, indicating that no cooperation between GNF-2 and AKIs in controlling the proliferation of the parental Ba/F3 cells was observed.

Next, the ability of GNF-2 to affect clonigenicity of Ba/F3 cells grown in the presence of IL-3 (10ng/ml) was examined. Imatinib and Dasatinib at 1μM exhibited minimal effect on clonigenicity of Ba/F3 cells. GNF-2 caused clonigenicity inhibition of Ba/F3, as shown in Figure S3. Moreover, presence of 1μM of Imatinib and Dasatinib marginally affected the IC_50_, arguing that no significant cooperation exists between GNF-2 and AKIs in inhibiting the proliferation and clonigenicity of the parental Ba/F3 cells.


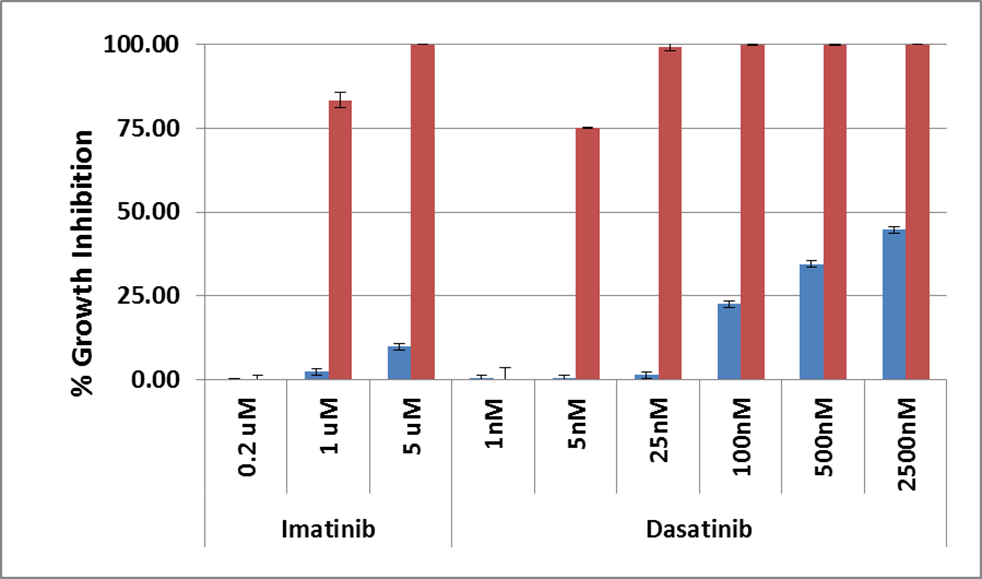


Figure S1: Effect of Imatinib and Dasatinib on the proliferation of Ba/F3 (Blue) and Ba/F3 p185 Bcr-Abl (Red) cells. The cells supplemented with 10 ng/ml of IL-3, were grown for 72 hrs in the presence of different Imatinib and Dasatinib concentrations. Cells were counted and percent of inhibition was calculated in relation to the solvent (0.5% DMSO) treated samples. Experiments were carried out in duplicates and repeated twice with comparable outcome.


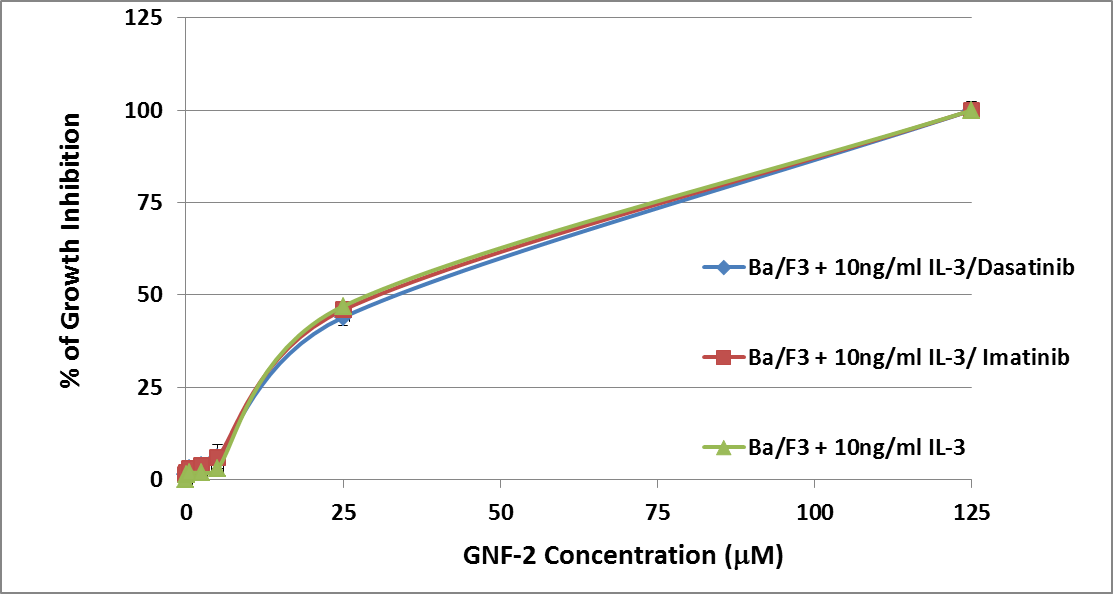


Figure S2: Cooperation between GNF-2 with Imatinib and Dasatinib in regulating proliferation of Ba/F3 cells. Ba/F3 supplemented with 10 ng/ml of IL-3 were grown for 72 hrs in the presence of various GNF-2 concentration (0.1, 0.5, 2.5, 5, 25 and 125 μM) alone or in combination with Imatinib (1 μM) and Dasatinib (50 nM). After 72 hrs incubation, cells were counted and percent of inhibition was calculated in relation to the solvent (0.5% DMSO) treated samples. Experiments were carried out in duplicates and repeated twice with comparable outcome.

Figure S3: Effect of GNF-2 and AKIs on the clonigenicity of Ba/F3 cells. Ba/F3 cells grown on soft agar were treated with solvent (DMSO 0.5%; Un), Imatinib (1µM), Dasatinib (1µM), and GNF-2 (5µM-100 µM) alone or in the presence of either 1µM Imatinib or 1µM Dasatinib. Experiments were carried out in duplicates and repeated twice with comparable outcome.
